# Sleep Disordered Breathing Severity Alters Overnight Temperature Changes in Children

**DOI:** 10.1111/jsr.70340

**Published:** 2026-03-29

**Authors:** Georgina Plunkett, Marisha Shetty, Maria Vaz‐Serra, Stephane Delanaud, Lauren C. Nisbet, Margot J. Davey, Gillian M. Nixon, Rosemary S. C. Horne, Veronique Bach

**Affiliations:** ^1^ Department of Paediatrics Monash University Melbourne Australia; ^2^ PeriTox (UMR_I 01), UPJV/INERIS Université de Picardie Jules Verne, CURS, Chemin du Thil Amiens France; ^3^ Melbourne Children's Sleep Centre Monash Children's Hospital Melbourne Australia

**Keywords:** obstructive sleep apnoea, paediatric sleep, peripheral skin temperature

## Abstract

The quality of sleep is very closely related to variations in body temperature. We aimed to assess the feasibility of skin temperature measurements in children attending the sleep laboratory for routine polysomnography and to determine whether there was a difference in body temperature between children with different severities of sleep disordered breathing. Skin temperatures were recorded using Thermochron iButtons (DS1922L, Maxim, Dallas Semiconductor Corp., USA) attached to the children's skin at three separate sites: chest (T_proximal_), the left and right feet. Distal skin temperature (T_distal_) was calculated as the average of temperatures for the left and right foot. Distal proximal gradient was calculated as T_distal_ minus T_proximal_. Sixty eight children were recruited and 63 (93%) children (24F/39M) aged 6 to 11.9 years with complete data were included in the study: 25 primary snoring, 28 Mild obstructive sleep apnoea, 10 Moderate/Severe obstructive sleep apnoea. In the children with primary snoring and Mild obstructive sleep apnoea distal temperature and distal proximal gradient exhibited typical distal vasodilation before sleep onset and progressive vasoconstriction after sleep onset, whereas there were no significant changes with time in the children with Moderate/Severe obstructive sleep apnoea. The rate of change of temperature before sleep onset to sleep onset was also significantly slower in the Moderate/Severe obstructive sleep apnoea group. Our study shows that temperature can be measured in children during overnight clinical polysomnography and that changes during sleep are altered in those children with Moderate/Severe obstructive sleep apnoea compared to children with milder sleep disordered breathing.

## 1 Introduction

The quality and quantity of sleep are closely related to variations in body temperature: falling asleep generally occurs in the period when there is a decrease in internal temperature while spontaneous morning awakening is associated with an increase in internal temperature (Campbell and Broughton 1994; Murphy and Campbell 1997). Prior to falling asleep there is a period of approximately 1.5 h during which distal vasodilation occurs, increasing the temperature in the feet and hands and thus dry heat losses from these distal areas to the environment, causing internal temperature to fall (van den Heuvel et al. 1998). The distal‐proximal temperature gradient (DPG) provides an estimation of peripheral vasodilation and is considered the most significant thermal marker and the best physiological determinant of sleep latency, sleep consolidation and overall sleep quality (Kräuchi et al. 2000). Several studies have pointed out that DPG and body temperature patterns differ from what is expected in various sleep disorders such as narcolepsy (Mayer et al. 1997) and insomnia (Lack et al. 2008).

Beyond a simple link between sleep and temperature regulation, induced variations in body temperature are associated with sleep in a causal manner, as has been shown by several studies in adults. Any practice that generates distal vasodilation, whether thermal (using a hot water bottle (Krauchi et al. 1999)), wearing a heating suit (Raymann et al. 2005) or socks (Ko and Lee 2018), or non‐thermal (turning off lights and lying down (Krauchi et al. 1997; Tikuisis and Ducharme 1996)) are capable of increasing sleepiness, reducing sleep latency and promoting sleep maintenance (Liao 2002; Van Someren et al. 2002). Conversely, when distal skin temperatures are reduced (e.g., when exposed to cold air or a cold bath), this promotes vigilance and wakefulness (Fronczek et al. 2008). Thus, from these observations, sleep or alertness could be improved in a non‐drug induced manner by simple thermal modifications without any side effects, as far as thermoregulatory responses to fight against cold or hot environment are not elicited.

Sleep, in terms of both quantity and quality, is of paramount importance for a child's neurological development and health. Despite differences in their sleep characteristics compared with adults, increased distal temperatures have been observed before sleep onset in preterm neonates in neonatal units (Bach et al. 2019) as well as in healthy school‐aged children (6–12 years) during recordings made at home (McCabe et al. 2018). Temperature changes in the home setting have been analysed in specific groups of children. In children with bipolar disorder, thermoregulatory dysfunction was associated with sleep onset difficulties (Murphy et al. 2014). In children with autism, atypical daily temperature patterns have been observed, with the slope flattening during afternoon and evening hours and an absent or subtle postprandial peak, compared to typically developing children (Martinez‐Cayuelas et al. 2024). In obese children, the percentage of rhythmicity, a marker of the robustness of wrist temperature, activity and position rhythm, and the amplitude, both components of the global circadian score, decreased with increasing obesity (Rodriguez‐Martin et al. 2024).

Sleep disordered breathing (SDB) is one of the most common disorders of sleep in children. SDB describes a range of severity of breathing disruption, from simple or primary snoring that is not associated with sleep disruption or gas exchange abnormalities at the mild end to obstructive sleep apnoea (OSA) at the severe end. Primary snoring affects up to 35% of children (Castronovo et al. 2003) while OSA occurs in 1%–6% of children (Marcus, Brooks, Draper, Gozal, Halbower, Jones, Schechter, Sheldon, et al. 2012a; Marcus, Brooks, Draper, Gozal, Halbower, Jones, Schechter, Ward, et al. 2012b). OSA results in increased effort of breathing to overcome the partial obstruction of the upper airway and has been demonstrated to be associated with increased metabolic rate and energy expenditure during sleep (Li et al. 2003). Heart rate variability analyses (Walter, Nixon, et al. 2013), analysis of the blood pressure and heart rate surges at respiratory event termination (Walter, Yiallourou, et al. 2013), and impaired blood flow in the limb (Imadojemu, Gleeson, Gray, et al. 2002; Imadojemu, Gleeson, Quraishi, et al. 2002), suggest impaired autonomic nervous system control and sympathetic hyperactivation. Given its role on distal vasomotricity, autonomic nervous system dysregulation has been suggested to explain the alterations of the distal temperature pattern in related to the severity of SDB in adults (Martinez‐Nicolas et al. 2017). This raises the question of the impact of SDB on body temperature patterns in children.

This study aimed to provide proof‐of‐concept data that there is a difference in skin temperature patterns during sleep in children and whether there are differences related to the severity of SDB. To our knowledge, this is the first study which aimed to assess the feasibility of skin temperature measurements in children attending the sleep laboratory for routine polysomnography (PSG) and to determine whether there was a difference in skin temperature patterns between children with different severities of SDB. We hypothesised that skin temperatures would be easily recorded with a high level (> 90%) of complete data across the night of a sleep study and that the body skin temperature pattern in relation to sleep would be different in children with Moderate/Severe OSA.

## 2 Methods

### 2.1 Subjects

Ethical approval for this study was obtained from the Monash Health Human Research Ethics Committee (04161C). Parents of typically developing children aged 6–12 years referred for assessment of sleep disordered breathing (SDB) between March and November 2023 were contacted prior to their child's sleep study and the study explained to them. If they consented for their child to participate, written informed consent was obtained from parents and verbal assent from the child when they attended the sleep laboratory.

### 2.2 Overnight Polysomnography

All children underwent an overnight PSG study attended by trained paediatric sleep scientists in accordance with guidelines established by the American Academy of Sleep Medicine (AASM) for paediatric sleep studies (Berry et al. 2020). Prior to the PSG study, height and weight were measured and used to calculate body mass index (BMI) *Z*‐scores. Obesity was defined as ≥ 95th percentile (BMI *z*‐scores > 1.65) and overweight as ≥ 85th percentile (BMI *z*‐scores > 1.04). Parents completed the Epworth Sleepiness Scale for Children and Adolescents (ESS‐CHAD) (Johns 2015). Routine hospital bedding was provided to all participants and information pertaining to the use of extra bedding, if the child wore socks for the duration of the PSG study and room temperature were also recorded.

Electrophysiological signals were recorded using a commercially available PSG system (Grael, Compumedics, Melbourne, Australia) with signals recorded at a sampling rate of 512 Hz. Electroencephalogram (EEG) (Cz, F3‐M2, F4‐M1, C3‐M2, C4‐M1, O1‐M2, O2‐M1), right and left electrooculogram (EOG), submental electromyogram (EMG), left and right anterior tibialis muscle EMG, and electrocardiogram (ECG). Respiratory characteristics were captured using abdominal and thoracic respiratory plethysmography (Pro‐Tech zRIP Effort Sensor, Pro‐Tech Services Inc., Mukilteo, WA), oronasal thermistor, nasal pressure and transcutaneous carbon dioxide (TcCO_2_), (Sentec, Therwil, Switzerland). Peripheral oxygen saturation (SpO_2_) was measured using Bitmos GmbH (Bitmos, Dusseldorf, Germany), which uses Masimo signal extraction technology for signal processing or Masimo Radical 7 (Masimo, Irving, CA), with both devices set to a 2‐s averaging time.

All PSG studies were scored manually in 30‐s epochs for sleep stages (N1, N2, N3 and REM) by trained paediatric sleep technicians using Compumedics ProFusion software. Respiratory events > 2 breaths in duration and arousals were scored according to AASM paediatric guidelines. Total sleep time (TST) was defined as the total time spent asleep, sleep latency as the time taken to reach the first epoch of sleep, and sleep efficiency as the percentage of TST given the time available for sleep. Wake after sleep onset (WASO) was defined as the time spent awake during the sleep period, and the arousal index as the total number of arousal events per hour of sleep. The obstructive apnoea hypopnoea index (OAHI), defined as the total number of obstructive apnoeas, mixed apnoeas and obstructive hypopnoeas per hour of TST, was used to define SDB severity. Children were grouped by SDB severity into primary snoring, which was defined as an OAHI ≤ 1 event/h, Mild OSA as an OAHI of > 1–≤ 5 events/h, and Moderate/Severe OSA as an OAHI of > 5 events/h. Other respiratory parameters included the central apnoea hypopnoea index (CAHI), defined as the number of central apnoeas and hypopnoeas per hour of TST, and the respiratory disturbance index (RDI), defined as the total number of obstructive, central and mixed apnoeas and hypopnoeas per hour of sleep. The SpO_2_ nadir was the lowest oxygen saturation point during TST, and the average SpO_2_ drop was the average drop in SpO_2_ that occurred following a respiratory event. The SpO_2_ ≥ 3% was defined as the number of times the SpO_2_ dropped by greater than or equal to 3% per hour of TST, SpO_2_ < 90% was defined as the number of times the SpO_2_ dropped below 90% per hour of TST, and the average transcutaneous pCO_2_ during TST.

### 2.3 Skin Temperature Measurements

Children's skin temperatures were recorded using Thermochron iButtons (DS1922L, Maxim, Dallas Semiconductor Corp., USA). The iButtons were pre‐set to record every 30 s to align with the epoch length used in PSG sleep staging and with high resolution (configuration mode of 0.0625°C in order to have a measurement step of 0.1°C). The iButtons were attached to the skin using air‐permeable adhesive skin tape in alignment with attachment methods reported in other studies of human skin temperature (Bach et al. 2020; Gompper et al. 2010; Krauchi et al. 2012; McCabe et al. 2018; Romeijn et al. 2012; van Marken Lichtenbelt et al. 2006). The iButtons were attached to the children's skin at three separate sites: chest (T_proximal_) and medial metatarsal areas of the left (T_left foot_) and right (T_right foot_) feet. Distal skin temperature (T_distal_) was calculated as the average of temperatures for the left and right foot, and the proximal skin temperature (T_proximal_) was sourced from the iButton applied to the chest. Distal proximal gradient (DPG) was calculated as T_distal_ minus T_proximal_. iButtons were attached prior to any PSG recording equipment in order to obtain the maximum amount of temperature data prior to sleep onset.

### 2.4 Data Analysis

PSG data were transferred via European Data Format to Labchart software (ADInstruments, Australia) and were separated into 30 s epochs according to sleep staging. Physiological parameters including heart rate, respiratory rate, and arterial saturation (SpO_2_) data were averaged for each 30 s epoch. The start and end time and physiological data for each epoch were exported to Excel for analysis. Each epoch was labelled with the appropriate sleep stage as determined by sleep scientists.

## 3 Statistical Analysis

Statistical analyses were conducted using SigmaPlot (Systat Software Inc. Version 14.5). Data were first tested for normality and equal variance. Demographic data, sleep and respiratory characteristics were compared between groups using a one‐way analysis of variance (ANOVA) if normally distributed or Kruskal Wallis one‐way ANOVA on Ranks if not, with appropriate post hoc tests conducted if differences were identified. Proportion data were analysed using a Chi‐squared test or Fisher's exact test where appropriate. To compare temperatures between subjects we designated sleep onset as time 0.

Temperature data were not normally distributed so the effects of time and group on skin temperature measure (proximal and distal temperature and DPG) were assessed using separate one‐way Kruskal Wallis one‐way ANOVA on Ranks for each SDB severity group across the night from 30 min prior to sleep onset to 6 h after sleep onset (−30 to +360 relative to sleep onset at *t* = 0). Six hours was selected as the cut‐off point as there was a significant increase in the number of participants with missing data after this time. For ease of interpretation, we examined the effects of time from the 30 min before sleep onset across the night using data averaged in 30‐min blocks. If significant differences were identified Dunn's multiple comparisons posthoc test with correction for multiple comparisons by controlling the false discovery rate was applied. To further characterise temperature changes with respect to SDB severity around sleep onset, linear regression was used to ascertain the rate of change of skin temperatures and DPG in the 30‐min period before and after sleep onset for each SDB group. Two‐way ANOVAs with Bonferroni posthoc tests were then used to analyse the effect of SDB severity, time window and the interaction of such factors on the rate of change. To identify if there were any relationships between temperature variability and sleep and respiratory characteristics we calculated the standard deviation of the temperature changes from sleep onset across the night (proximal, distal and DPG) in each child and conducted correlation analyses with the OAHI, SpO_2_ nadir, RDI, arousal index, sleep efficiency and ESS‐CHAD. In addition, we conducted correlation analysis between temperature variability (proximal, distal and DPG) from the 30 min before sleep onset to sleep onset and sleep latency. All data are presented as median and interquartile range (IQR) as a majority of the data were not normally distributed. A *p*‐value of < 0.05 was accepted as statistically significant.

## 4 Results

Sixty eight children referred to Melbourne Children's Sleep Centre for assessment of SDB were recruited for this study; 1 child was excluded due to software failure, 1 due to non‐compliance and 3 were excluded as > 1 iButton came off during the study leaving 63 (93%) children (24 female, 39 male) aged 6 to 11.9 years with complete data included in the study. Children were divided by SDB severity based on the OAHI: 25 in the PS group, 28 in the Mild OSA group and 10 in the Moderate/Severe OSA group. Demographic data are presented in Table 1. There were no differences between the groups for age, or BMI *z*‐score, % overweight or obese, room temperature or ESS‐CHAD scores.

**TABLE 1:** Demographic characteristics for primary snorers, mild OSA and moderate/severe OSA.

	Primary snorers	Mild OSA	Moderate/severe OSA
Number	25	28	10
Sex	10 F/15 M	10 F/18 M	4 F/6 M
Age (years)	7.8 (6.5–9.7)	7.6 (6.9–9.5)	7.9 (7.4–10.4)
BMI *z*‐score	0.7 (−0.4–1.5)^a^	0.9 (0.1–1.5)	0.5 (0.2–1.9)
% obese	20.8^a^	14.3	30.0
% overweight	20.8^a^	28.6	0
Room temperature (°C)	23.0 (22.7–23.5)^a^	23.0 (22.3–23.1)^b^	22.9 (22.4–23.1)
ESS‐CHAD	5.0 (2.0–9.0)^b^	5.0 (3.0–7.8)	5.0 (3.5–7.5)^c^

*Note:* Values are median and IQR.

Abbreviations: BMI, body mass index; ESS‐CHAD, Epworth sleepiness scale‐children and adolescents.

^a^ Number of children = 24.

^b^ Number of children = 23.

^c^ Number of children = 9.

### 4.1 Sleep and Respiratory Characteristics

Sleep characteristics for the groups are presented in Table 2. The children with Moderate/Severe OSA had longer sleep latency and spent more time in N1 sleep (*p* < 0.05 for both) compared with the PS group. Sleep efficiency was lower in the Moderate/Severe OSA and Mild OSA groups compared with the PS group (*p* < 0.05 for both). There were no differences between the groups for time in bed, sleep period time, total sleep time, REM latency, time spent in N2, N3, NREM and REM sleep and frequency of periodic leg movements.

**TABLE 2:** Sleep characteristics of children with primary snoring, mild OSA and moderate/severe OSA.

	Primary snorers	Mild OSA	Moderate/severe OSA
Number	25	28	10
Time in bed (min)	511 (494–521)	513 (498–543)	521 (482–558)
Sleep period time (min)	489 (477–503)	480 (440–509)	496 (439–523)
Total sleep time (min)	456 (431–482)	434 (368–475)	419 (362–467)
Sleep latency (min)	17 (8–30)	18 (13–40)	32 (23–39)^a^
REM latency (min)	159 (99–183)	162 (138–192)	152 (126–182)
Sleep efficiency (%)	90 (85–93)	86 (71–90)^b^	80 (74–90)^a^
Wake after sleep onset (%SPT)	5 (4–10)	7 (3–20)	14 (4–21)
N1 %TST	5 (4–7)	7 (5–4)	8 (7–12)^a^
N2 %TST	45 (43–51)	46 (40–50)	43 (36–45)
N3 %TST	28 (23–32)	28 (22–33)	28 (25–35)
NREM Sleep %TST	81 (77–83)	80 (77–84)	81 (79–85)
REM Sleep %TST	18 (16–23)	20 (16–22)	19 (14–20)
PLM (TST events/h)	0 (0–1)	1 (0–2)	0 (0–2)

*Note:* Values are median and IQR.

Abbreviations: NREM, non‐rapid eye movement sleep; PLM, periodic leg movements; REM, rapid eye movement sleep; TST, total sleep time.

^a^ *p* < 0.05 primary snorers versus moderate/severe OSA.

^b^ *p* < 0.05 primary snorers versus mild OSA.

Respiratory characteristics are reported in Table 3. By design, OAHI and RDI were significantly different between all groups. Similarly, children with Moderate/Severe OSA had a higher arousal index compared to those in the Mild OSA and PS groups. REM RDI and SpO_2_ > 3% drop were greater in the Moderate/Severe OSA and Mild OSA groups compared with the PS group. There were no differences between the groups for CAHI, SpO_2_ nadir, average SpO_2_ drop, SpO_2_ < 90% and average transcutaneous CO_2_.

**TABLE 3:** Respiratory characteristics for children with primary snoring, mild OSA and moderate/severe OSA.

	Primary snorers	Mild OSA	Moderate/severe OSA
Number	25	28	10
OAHI (events/h)	0.4 (0.1–0.6)	3.1 (1.6–3.3)^c^	9.15 (7.5–20.6)^a^, ^d^
CAHI (events/h)	1.0 (0.7–1.7)	1.4 (0.6–2.1)	1.7 (0.4–2.4)
RDI (events/h)	1.5 (1.1–2.5)	4.1 (3.4–4.9)^c^	11.5 (7.8–23.1)^a^, ^e^
Arousal Index (events/h)	9.6 (7.9–12.4)	11.6 (9.9–12.7)	14.1 (12.3–16.6)^a^, ^d^
REM RDI (events/h)	2.2 (0.6–4.3)	6.0 (4.1–9.1)^c^	16.7 (5.5–29.1)^a^
SpO_2_ Nadir (%)	93.0 (91.3–94.0)	93.0 (91.3–94.0)	91.5 (87.0–93.0)
Average SpO_2_ Drop (%)	4.0 (3.0–4.0)	4.0 (3.0–4.0)	4.0 (3.0–4.0)
SpO_2_ < 90% (events/h)	0.0 (0.0–0.0)	0.0 (0.0–0.0)	0.0 (0.0–0.2)
SpO_2_ > 3% Drop (events/h)	1.2 (0.6–1.9)	2.1 (1.2–3.0)^b^	8.1 (1.6–12.0)^a^
Average TcCO_2_ TST (mmHg)	42.4 (40.2–43.8)	41.4 (39.5–42.9)	41.1 (37.9–43.7)

*Note:* Values are median and IQR.

Abbreviations: CAHI, central apnoea hypopnea index; OAHI, obstructive apnoea hypopnea index; RDI, respiratory disturbance index; SpO_2_, oxygen saturation; TST, total sleep time.

^a^ *p* < 0.001 primary snorers versus moderate/severe OSA.

^b^ *p* < 0.05 primary snores versus mild OSA.

^c^ *p* < 0.001 primary snorers versus mild OSA.

^d^ *p* < 0.05 mild OSA versus moderate/severe OSA.

^e^ *p* < 0.01 mild OSA versus moderate/severe OSA.

### 4.2 Temperature Changes Across the Night in the Different SBD Groups

Temperature data for two subjects was removed for the following reasons: (1) Proximal temperature and by default, DPG data prior to lights out were removed for one subject with PS as the chest button was placed later than the feet buttons; (2) All proximal and distal temperature and DPG data were removed for one subject with Moderate/Severe OSA until sleep onset as they left the bed several times.

**FIGURE 1:**
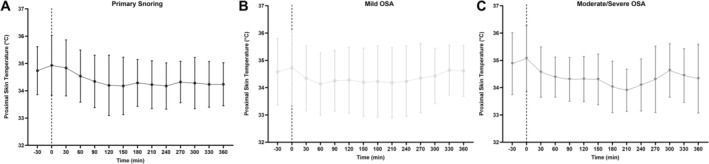
The effect of time from the 30 min before sleep onset (time 0) across the night on mean proximal skin temperature (°C) in the Primary Snoring (A), Mild OSA (B) and Moderate/Severe OSA (C). Values are median and IQR.

**FIGURE 2:**
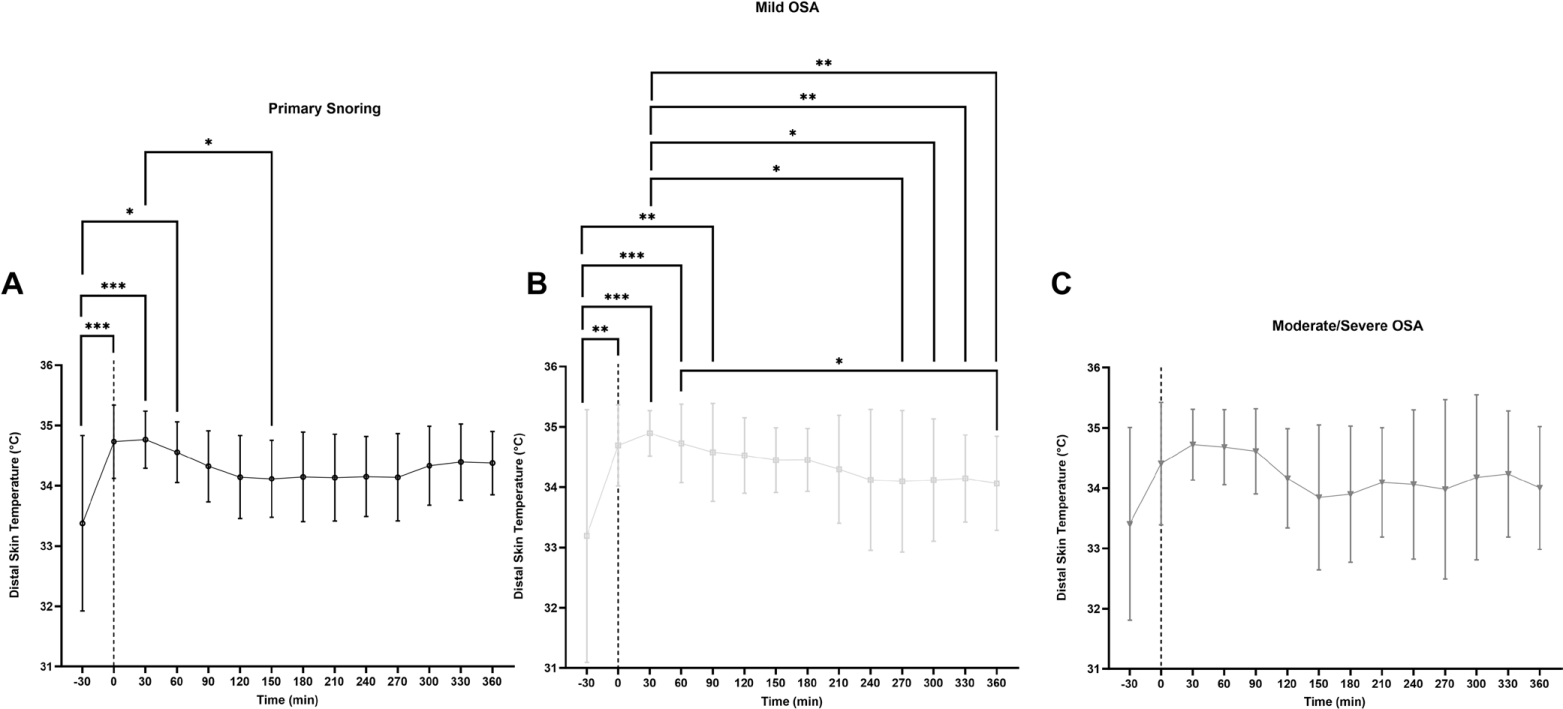
The effect of time from the 30 min before sleep onset (time 0) across the night on mean distal skin temperature (°C) in the Primary Snoring (A), Mild OSA (B) and Moderate/Severe OSA groups (C). Values are median and IQR. *p < 0.05; **p < 0.01; ***p < 0.001.

**FIGURE 3:**
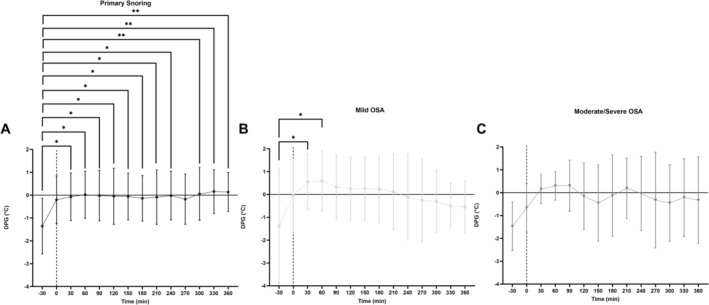
The effect of time from the 30 min before sleep onset (time 0) across the night on mean DPG (°C) in the Primary Snoring (A), Mild OSA (B) and Moderate/Severe OSA (C) groups. Values are median and IQR. *p < 0.05 and **p < 0.01.

The effect of time across the night is shown for each SDB severity group separately in Figure 1 for proximal skin temperature, Figure 2 for distal skin temperature and Figure 3 for DPG. There were no effects of time in any group on proximal skin temperature (Figure 1). For distal skin temperature in the PS group (Figure 2A), temperatures were significantly lower before sleep onset compared to sleep onset, +30 and +60, and significantly higher at +30 compared to +150 min after sleep onset. In the Mild OSA group (Figure 2B), distal skin temperature was significantly lower before sleep onset compared to sleep onset, +30, +60 and +90, higher at +30 compared to +270, +300, +330 and +360 and higher at +60 compared to +360 min after sleep onset (Figure 2B). In the Moderate/Severe group, there were no significant changes in distal skin temperature at any time across the night (Figure 2C). For DPG in the PS group, DPG was significantly lower before sleep onset compared to all other time points beside +270 (Figure 3A). For the Mild OSA group, DPG was significantly lower before sleep onset compared to sleep onset +30 and +60 min after sleep onset (Figure 3B). In the Moderate/Severe group, there are no significant differences in DPG between any time point across the night (Figure 3C).

### 4.3 Rate of Change in Temperature Before and After Sleep Onset

**FIGURE 4:**
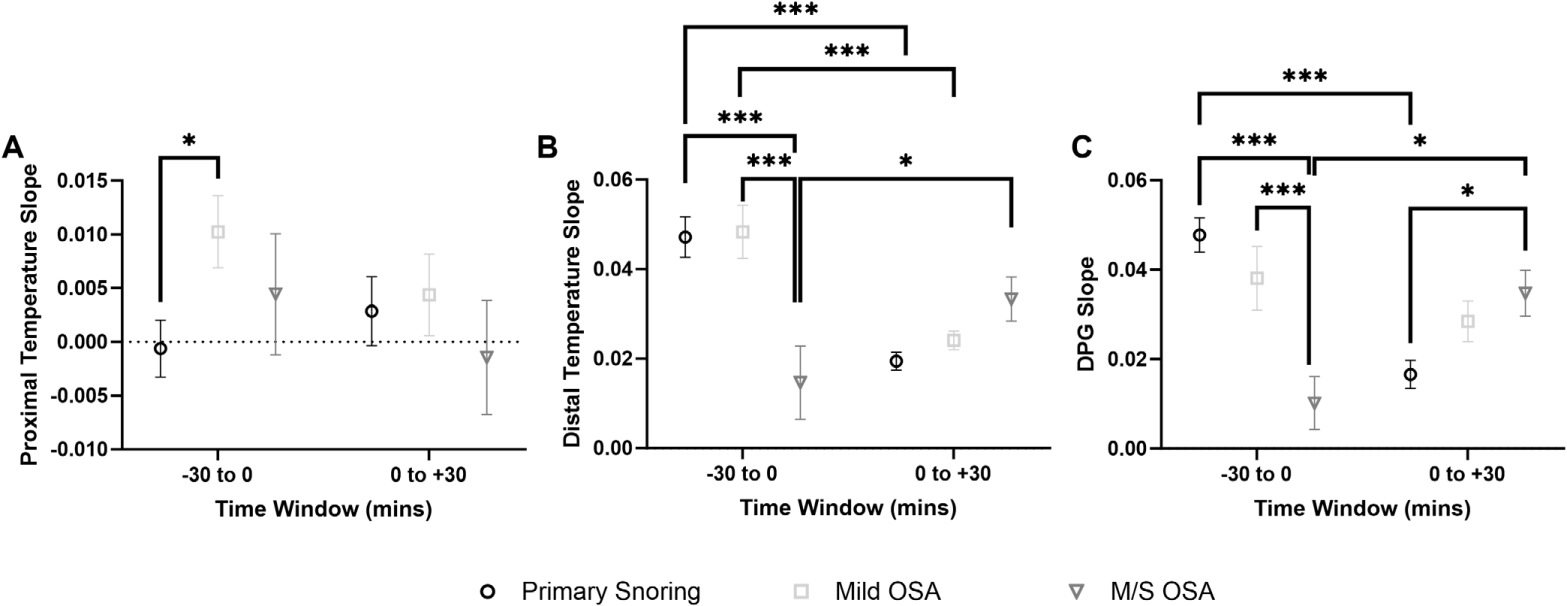
Rate of change for (A) proximal and (B) distal skin temperatures and (C) distal to proximal temperature (DPG) (°C/min) for the 30 min before sleep onset (−30 to sleep onset time 0) and the 30 min after sleep onset (0 to +30) in the children with Primary snoring, Mild OSA and Moderate/Severe OSA. Values are mean and SEM. *p < 0.05; **p < 0.01; ***p < 0.001.

The rate of change in proximal and distal skin temperatures and DPG for the 30 min before sleep onset (−30 to 0) and the 30 min after sleep onset (0 to +30), expressed as °C/min, is presented in Figure 4. The change in proximal skin temperatures (Figure 4A) was significantly faster in the Mild OSA (0.01°C/min) vs the PS group (−0.0006°C/min, *p* < 0.05) before sleep onset. No differences were identified between the SDB groups after sleep onset.

The rate of distal skin temperature change (Figure 4B) differed significantly according to time. The distal skin temperature increased significantly faster before vs after sleep onset for both the PS (0.047°C/min vs 0.019°C/min, *p* < 0.001) and Mild OSA groups (0.048°C/min vs 0.024°C/min, *p* < 0.001). In contrast, in the Moderate/Severe OSA group, the rate of distal temperature change was significantly lower before sleep onset compared with after (0.015°C/min vs 0.033°C/min, *p* < 0.05).

When comparing within the time windows, the SDB group was found to have an effect on distal skin temperature rate of change. Before sleep onset, the rate of change in temperature was significantly slower in the Moderate/Severe group (0.015°C/min) compared to both the PS (0.047°C/min) and Mild OSA groups (0.048°C/min, *p* < 0.001 for both). There were no differences in distal slope between the SDB groups during the 0 to +30 time window.

DPG rate of change (Figure 4C) was also significantly affected by both SDB group and time. Before sleep onset, DPG change was significantly slower in the Moderate/Severe OSA group (0.010°C/min) compared to the PS group (0.048°C/min, *p* < 0.001) and the Mild OSA group (0.038°C/min, *p* < 0.001). However, after sleep onset, the rate of DPG change was significantly faster in the Moderate/Severe OSA (0.033°C/min) vs the PS group (0.019°C/min, *p* < 0.05). Within the PS group, DPG varied significantly faster before vs after sleep onset (0.048°C/min vs 0.019°C/min, *p* < 0.001). In contrast, DPG slope was significantly lower prior to sleep onset compared to after sleep onset in the Moderate/Severe group (0.010°C/min vs 0.033°C/min, *p* < 0.05). There were no significant differences in rate of change of DPG between the time windows before and after sleep onset in the Mild OSA group.

## 5 Correlation Between Temperature Variability and Sleep and Respiratory Characteristics

Results of correlation analysis between temperature variability and sleep and respiratory characteristics are presented in Table 4. In the group as a whole, we identified that distal temperature variability was negatively correlated with SpO_2_ nadir (−0.257, *p* < 0.05) and positively correlated with ESS‐CHAD scores (*r* = 0.260, *p* < 0.05), while DPG variability was positively correlated with OAHI (*r* = 0.289, *p* < 0.05) and RDI (*r* = 0.336, *p* < 0.01). We did not find any relationship between temperature variability before sleep onset and sleep onset latency.

**TABLE 4:** Correlation analysis between temperature variability and sleep and respiratory characteristics.

	Proximal skin temperature		Distal skin temperature		DPG	
	*r*	*p*	*r*	*p*	*r*	*p*
OAHI	0.093	0.469	0.081	0.527	0.289	**0.022**
SpO_2_ nadir	−0.057	0.659	−0.257	**0.044**	−0.161	0.210
RDI	0.165	0.197	0.052	0.686	0.336	**0.007**
Arousal index	−0.0316	0.805	0.105	0.410	0.0350	0.785
Sleep efficiency	0.121	0.344	−0.020	0.875	−0.087	0.495
ESS‐CHAD	−0.130	0.320	0.260	**0.045**	0.053	0.687
Sleep latency	0.040	0.756	0.001	0.995	−0.034	0.790

*Note:* OAHI, SpO_2_ nadir, RDI, Arousal Index, sleep efficiency and ESS‐CHAD correlations used variability of temperatures during overnight sleep and sleep latency used variability of temperature before sleep onset. These are the *p* values and we have simply highlighed the significant values by bolding them.

Abbreviations: ESS‐CHAD, Epworth sleepiness scale‐children and adolescents; OAHI, obstructive apnoea hypopnea index; RDI, respiratory disturbance index; SpO_2_, oxygen saturation.

## 6 Discussion

In this study we aimed to assess the feasibility of skin temperature measurements in children attending the sleep laboratory for routine polysomnography and to determine whether there was a difference in body temperature patterns between children with different severities of SDB. We found that measuring skin temperature in children during a routine clinical overnight sleep study was feasible, with 93% of studies providing usable data. We identified that the Moderate/Severe SDB group had flattened skin temperatures and DPG patterns whereas distal temperature increased from the 30 min before sleep onset to sleep onset (time 0) in the children with PS and Mild OSA, and then progressively declined.

This was the first study to our knowledge to measure skin temperatures in children during routine clinical sleep studies. We did not identify any adverse effects of using the iButtons and usable data was obtained from 93% of studies. Previous studies have used the same Thermochron iButtons to measure temperature in children during sleep in the home and found that measures of skin temperature during sleep were practical for children in their home settings with 94% of recordings providing usable data (McCabe et al. 2018). To minimise the number of iButtons placed on the skin we only used 1 proximal iButton placed on the chest and 2 distal iButtons placed on the feet with the data averaged for these, rather than the 8 iButtons used in the study by McCabe et al. (2018).

Skin temperatures and DPG patterns analysed from 30 min before sleep onset to 6 h after sleep onset were altered in those children with Moderate/Severe OSA compared to children with milder SDB. In the PS and in the Mild OSA groups, distal temperature and DPG patterns are consistent with healthy adults' results and same‐age children's results (Krauchi 2007; McCabe et al. 2018). Distal vasodilation occurs before sleep onset (similar in both PS and Mild groups as indicated by the slope values, Figure 4, panels B and C) and is followed by a progressive distal vasoconstriction after sleep onset (Krauchi 2007; McCabe et al. 2018). This vasodilation seems to be more progressive and longer in the Mild OSA group. Pre‐sleep vasodilation has been attributed to a change in posture with lying down, a rise in melatonin associated with lights off and an increase in ambient temperature associated with being in bed (Krauchi 2007). Distal vasodilation allows the achievement of a ‘completely relaxed, one‐compartment body’ state (Krauchi and Deboer 2010) where skin and internal temperatures are equal (DPG = 0). As a result, disappearance of the thermoregulatory shell of the body (adults: (Krauchi and Deboer 2010)) and homogenisation of the different local skin temperatures (preterm neonates: (Barcat et al. 2017)) reduce the thermal inputs from the skin to the hypothalamus and increase sleep propensity. DPG reaches the 0°C plateau as soon as +30 min in the PS group, whereas DPG reaches positive values (higher distal compared to proximal temperature) from 30 to 90 min, with a progressive decline to negative DPG values until the end of the 360 min period in the Mild OSA group. We have previously shown that the larger amplitude of the vasodilation before sleep onset was correlated with more rapid sleep onset in preterm neonates (Barcat et al. 2017). Consistent with these results, it is interesting to notice that both the PS and Mild OSA groups exhibit similar sleep latencies (median values of 17 and 18 min, respectively). To investigate this further we conducted correlation analysis between temperature variability (proximal, distal and DPG) from the 30 min before sleep onset to sleep onset and sleep latency, but did not identify any relationships. As expected, proximal temperature variations with time did not reach statistical significance, since the levels and patterns of proximal skin temperature are intermediate, between the distal (with many arteriovenous anastomoses, (Bergersen 1993)) and the core internal temperature.

In contrast, the Moderate/Severe group looks very different, with an absence of skin temperature rhythms (proximal, distal and DPG, Figures 1, 2, 3), very small vasodilation before sleep onset (as distal temperature and DPG slopes are close to 0, Figure 4, panels B and C) and large inter‐individual variability especially in distal temperature and DPG. Vasodilation seems to be amplified or postponed after the sleep onset with increased distal and DPG slopes when compared to pre‐sleep onset slopes. This could be related to difficulty in falling asleep, as indicated by significantly longer sleep latency (median of 32 min vs. 17–18 min), and higher N1% (8 vs. 5% for the PS group). Other sleep characteristics, except sleep efficiency, did not significantly differ between the three groups, consistent with our previous work showing that sleep architecture using conventional measures seems to be relatively preserved in children with SDB in contrast to adults, despite neurocognitive and behavioural impairments (Yang et al. 2010), suggesting that conventional sleep analyses may not be sensitive enough to detect sleep disturbances in SDB. Consistent with an adult study (Martinez‐Nicolas et al. 2017), temperature pattern impairment in our Moderate–Severe group was not related to increased daytime sleepiness assessed with the ESS‐CHAD (Table 1), however distal temperature variability was positively correlated with ESS‐CHAD scores in the group as a whole. The children in the Moderate/Severe group aroused more frequently during sleep, as indicated by the elevated arousal index, and this could disrupt their temperature regulation. Even without a cortical arousal, the termination of either an apnoea or a hypopnoea is associated with a surge in heart rate and blood pressure, which is similar in magnitude in children compared to adults (O'Driscoll et al. 2009), suggesting impaired autonomic nervous system control and sympathetic hyperactivation (Walter, Nixon, et al. 2013; Walter, Yiallourou, et al. 2013). In adults with SDB compared to healthy controls, Martinez‐Nicolas et al. (2017) have suggested a hypothetical role of SDB in the disruption of the circadian system function and observed unstable, flattened and phase‐advanced distal wrist skin temperature. They observed that these alterations were greater in the most severe group (AHI > 30 events/h) compared to the pooled Primary Snoring and Mild patients (AHI < 15 events/h). Garcia‐Molina et al. have also demonstrated reduced distal temperature variability during the night in adults with a higher AHI (Molina et al. 2025). Our results with flattened distal temperature and DPG patterns are quite consistent in school‐aged children with Moderate to Severe SDB (despite different AHI levels), but we observed that vasodilation seems to be postponed after sleep onset in children, whereas Martinez‐Nicolas et al. (2017) observed phase‐advance pattern in adults. They also hypothesised that lower distal temperature could result from the higher sympathetic activation (and as a result higher vasoconstriction) due to respiratory events during sleep.

Both Martinez‐Nicolas et al. (2017) in adults and now our present study in children reinforce the relationship between SDB and skin temperature patterns. From a clinical point of view, it is important to stress that flattened or reduced amplitudes of the circadian rhythm are associated with and could be markers of aging and increased mortality (Myers and Badia 1995; Riemersma‐van der Lek et al. 2008; Rodriguez‐Martin et al. 2024; Zuurbier et al. 2015). Interestingly, DPG and skin temperature patterns may recover when continuous positive airway pressure is used to treat OSA in adult patients (Martinez‐Nicolas et al. 2017) as does vascular function (Imadojemu, Gleeson, Quraishi, et al. 2002). This is still to be analysed in children.

Our study was not without limitations. We must acknowledge that the number of children in the Moderate/Severe group was small; however, this reflects that seen in clinical practice where the majority of children referred for a sleep study have primary snoring or Mild OSA. In addition, we acknowledge that we did not have a non‐snoring control group as the children in this study were all referred for assessment of SDB and we did not adjust for multiple testing.

In conclusion, our study has shown that temperature can be measured in children during an overnight clinical PSG and that skin temperatures and DPG patterns in the Moderate/Severe group contrast to mild SDB groups: patterns are flattened without vasodilation before sleep onset that is postponed after sleep onset.

## Author Contributions

**Georgina Plunkett:** investigation, formal analysis, writing – review and editing. **Marisha Shetty:** investigation, formal analysis, writing – review and editing. **Maria Vaz‐Serra:** formal analysis, writing – review and editing. **Stephane Delanaud:** methodology, writing – review and editing. **Lauren C. Nisbet:** resources, writing – review and editing. **Margot J. Davey:** resources, writing – review and editing. **Gillian M. Nixon:** resources, writing – review and editing. **Rosemary S. C. Horne:** funding acquisition, supervision, writing – original draft preparation, writing – review and editing. **Veronique Bach:** conceptualisation, funding aquisition, writing – original draft preparation, writing – review and editing.

## Funding

This work was supported by the Hubert Curien Partnership (47184NA) and the National Health and Medical Research Council (1195453).

## Conflicts of Interest

The authors declare no conflicts of interest.

## Data Availability

The data that support the findings of this study are available from RSC Horne but restrictions apply to the availability of these data, which were used under licence for the current study, and so are not publicly available. Data are however available from the authors upon reasonable request and with permission of RSC Horne.
